# Parliament and the Profession

**Published:** 1916-08-12

**Authors:** 


### PARLIAMENT AND THE PROFESSION.

### Industrial Poisoning.

Replying to Mr. Anderson, Mr. Brace stated that there had unfortunately been a considerable increase in the number of cases of anthrax reported under the Factory and Workshops Act for the six months ending June 1916. The increase could only be accounted for by the fact that owing to the heavy demand manufacturers had been compelled to use all kinds of material. The problem of prevention was being thoroughly investigated by a Departmental Committee, and there was good reason to hope that they would be able to put forward effective proposals. As regards poisoning from dopes containing tetrachlorethane, this danger should very shortly be eliminated altogether by the substitution of the non-poisonous dopes now being brought into use.

### Tuberculous Recruits.

An important statement with regard to the measures taken to prevent tuberculous persons entering the Army was made by Mr. Forster in reply to a question. Mr. Anderson had asked whether he was aware that numbers of tuberculous persons were being taken into the Army; that in a large number of cases these consumptive soldiers, without value from a military standpoint and an additional burden to the State, were at the same time a source of danger to healthy soldiers; whether his attention had been called to the statement of the Leeds Insurance Committee, that out of 110 tuberculous insured persons who enlisted in Leeds during the year they had lost trace of sixty-six, but that out of the remaining forty-four about thirty had had to be discharged, while three had died; and whether it was proposed to take any action in this matter. In his reply Mr. Forster said he was not aware that considerable numbers of tuberculous persons were being taken into the Army, but he agreed with Mr. Anderson that no such persons should be taken. Instructions had been issued to recruiting medical boards to ask each man definitely whether he had been under treatment in a sanatorium, and whether his name had been notified to the sanatorium authorities as suffering from consumption. No man whose answer to either of these questions was in the affirmative would be taken, but it would obviously be necessary to verify his statements. The Local Government Board had also arranged for local tuberculosis officers to supply particulars of all men of military age who are on the tuberculosis registers, and these particulars would be notified to the proper recruiting areas, with instructions that such men were not to be accepted for service. The advice of these tuberculosis officers would also be asked for in cases of doubt.

For Matrons' and Sisters' Appointments see p. 460.

